# Epstein–Barr virus infection and genome polymorphisms on gastric remnant carcinoma: a meta-analysis

**DOI:** 10.1186/s12935-020-01498-z

**Published:** 2020-08-18

**Authors:** Chao Lu, Hongtao Zhang, Weihua Zhou, Xingyong Wan, Lan Li, Chaohui Yu

**Affiliations:** Department of Gastroenterology, The First Affiliated Hospital, College of Medicine, Zhejiang University, 79 Qingchun Road, Hangzhou, 310003 China

**Keywords:** Epstein–Barr virus, Gastric remnant carcinoma, Clinicopathologic characteristics, Genotypes, EBV associated GRC

## Abstract

**Background:**

Previous studies reported that Epstein–Barr virus (EBV) may play a causal role in the pathogenesis of gastric remnant carcinoma (GRC). However, there was still some controversy.

**Methods:**

Articles published until July 15, 2020, in PubMed, MEDLINE, Embase and CNKI databases were selected. According to the inclusion criteria, corresponding data of included articles were abstracted and used for statistical analysis.

**Results:**

Thirteen papers were finally enrolled, nine of which showed the result that the risk of EBV infection rate in the GRC was higher than conventional gastric carcinoma (OR = 5.22, 95% CI 3.89–7.00). In addition, we found that EBV associated GRC (EBVaGRC) had higher rate of Billroth-II (OR = 3.80, 95% CI 1.90–7.57), carcinoma in anastomotic site (OR = 2.41, 95% CI 1.27–4.56) and diffuse type (Lauren classification) (OR = 1.97, 95% CI 1.04–3.73),while sex, initial diagnosis and lymphocytic infiltration were calculated no statistical difference. By genetic polymorphism analysis, “V-val” subtype of EBNA1 (OR = 21.84, 95% CI 11.92–31.76) and “C” subtype of BamHI-W1/I1 (OR = 7.07, 95% CI 1.47–34.03) were observed to be highly expressed in EBVaGRC.

**Conclusion:**

EBV infection rate in the GRC was higher. Further analysis showed that Billroth-II, carcinoma in anastomotic site and diffuse type (Lauren classification) were associated to EBVaGRC. Through analysis of EBV genome polymorphisms, we thought that “V-val” subtype of EBNA1 and “C” subtype of BamHI-W1/I1 may become predictor of EBVaGRC.

## Background

Gastric remnant carcinoma (GRC) is defined as a carcinoma occurring in the gastric stump 5 or more years after distal gastric resection for benign disease such as gastric ulcer and duodenal ulcer or gastric malignancy [1, 2]. The etiology and pathogenesis associated with malignant transformation in the gastric stump are poorly understood.

An oncogenic association between gastric carcinoma (GC) and Epstein–Barr virus (EBV) is well known [3]. EBV-associated-gastric carcinoma (EBVaGC) prevalence varied from 2 to 18% in different countries [4]. Lee et al. have reported a meta-analysis indicating that the clinicopathological and molecular characteristics of EBVaGC were quite different from those of conventional gastric adenocarcinoma [5]. As early as 1994, Yamamoto et al. have published the relationship between GRC and EBV, which revealed that the EBV may play an important role in the carcinogenesis of GRC [6].

EBV is a gamma herpesvirus, which is a ubiquitous cause of infection in humans worldwide. Evidence of prior infection is present in adults throughout the world, with over 90% showing a serologic response [7]. In addition to GC, EBV is also closely related to Burkitt’s lymphoma (BL), Hodgkin lymphoma (HD) and nasopharyngeal carcinoma (NPC) [8–10]. The exact role of EBV in the pathogenesis of EBV-associated-gastric remnant carcinoma (EBVaGRC) remains to be determined. Previous study has reported that different genotype EBV expressed in GRC, which may play a key role in the pathogenesis of EBVaGRC [11]. However, there were still some controversies.

In this study, we aimed to explore whether there was a higher EBV infection rate in GRC compared to conventional GC (CGC). The exploration of related clinicopathologic factors such as Lauren classification between EBVaGRC and EBV-negative-gastric remnant carcinoma (EBVnGRC) were also one of the purposes in the study. In addition, we also analyzed EBV genome polymorphisms in EBVaGRC in order to reveal some relevance.

## Materials and methods

### Data selection and data abstraction

We searched articles published until July 15, 2020, in the PubMed, MEDLINE and Embase databases, and also searched Chinese database named CNKI. In order to cover as many articles as possible, following terms were used to search: *((residual gastric cancer) OR (residual gastric carcinoma) OR (gastric remnant carcinoma) OR (gastric remnant cancer) OR (gastric stump cancer) OR (gastric stump carcinoma)) AND ((Epstein*–*Barr virus) OR EBV OR (EB virus) OR EBNA1 OR EBNA2 OR EBNA3C OR (*30 bp deletion in *LMP1) OR BamHI*-*F OR BamHI*-*W1/I1)*. EBNA1, EBNA2, EBNA3C, 30 bp deletion in LMP1, BamHI-F and BamHI-W1/I1 are all EBV genotypes. After the primary election, the inclusion and exclusion criteria were used for the next screening. Articles would be included if they met all the following criteria: (1) Published as full articles; (2) Related data of GRC and EBV can be calculated; (3) The initial malignant tumor was cured before GRC; (4) EBV positivity was identified by in situ hybridization; (5) No other coexisting malignant tumors. Articles which did not meet the above criteria or duplicate publication were excluded.

Main study characteristics for analysis such as EBVaGRC data, EBV genotypes data were extracted to Microsoft Excel (2019 edition; Microsoft, Redmond, WA, USA) for effective organization. In addition, clinical features such as reconstruction style, initial diagnosis, location of GRC, Lauren classification, sex of patients, lymphocytic infiltration between EBVaGRC and EBVnGRC were also abstracted to assess risk factors.

Studies were independently screened by title and abstract by CL and XY. Both authors subsequently performed full articles. If there was a disagreement, XYW would evaluate the disagreement again and form a final result after the trade. Risk of bias and Quality assessment were assessed using elements of the STROBE checklist for studies included [12]. Once an overall gene effect was confirmed, the genetic model-free approach was used to estimate the genetic effects and mode of inheritance [13]. In addition, the work was conducted in accordance with the Preferred Reporting Items for Systematic Reviews and Meta-Analyses (PRISMA) statement [14].

### Statistical analysis

The odds ratio (OR) combined 95% confidence intervals (CIs) was used to describe dichotomous variable, which contained EBVaGRC data, EBVnGRC data, clinical features and genome polymorphisms data. For individual studies of genome polymorphisms with no events in one or both groups, a continuity correction of 0.5 would be added to each cell for each effect measure, as implemented in Review Manager 4.2 [15], and OR was used to describe it. Statistical heterogeneity was assessed by the I^2^ statistic and Cochran’s Q-test. Then pooled estimates were obtained using the fixed-model (Mantel and Haenszel) method (if I^2^ ≤ 50%, P > 0.1) or random-model (M-H heterology) method (if I^2^ > 50%, P ≤ 0.1) [16]. What we need to explain here was that random-model method was more widely used. Regardless of heterogeneity of pooled estimates, random-model method can be selected. When heterogeneity was high, random-model method was the only choice. While heterogeneity was low, both random-model and fixed-model model can be selected. In this study, fixed-model model was selected [16]. Furthermore, we used sensitivity analysis, which was conducted by metainf application, to evaluate whether the meta-analysis results were stable and reliable. Metainf investigated the influence of a single study on the overall meta-analysis estimate. And this command showed graphically the results of an influence analysis. Publication bias was estimated by Begg’s test (P > 0.05 suggests no publication bias). All analyses were carried out through the application of STATA 15 (StataCorp., College Station, TX, USA).

## Results

### Basic characteristics

**Fig. 1:**
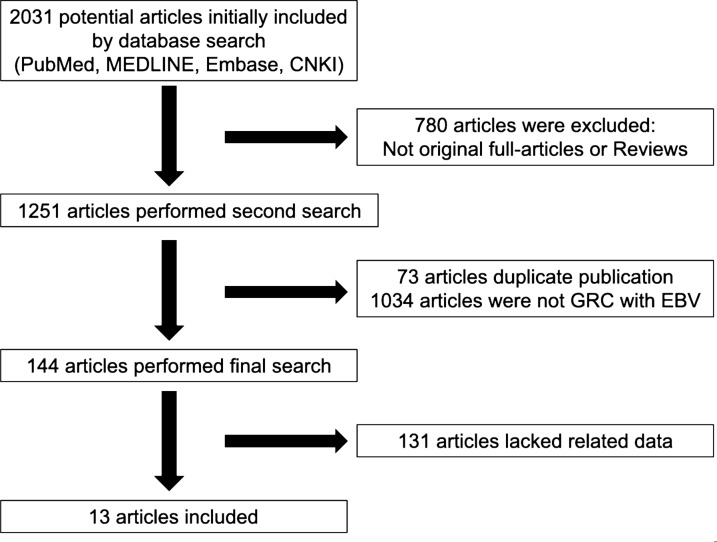
Selection flowchart

Our search identified 2031 related articles, of which 13 papers finally enrolled according to the inclusion criteria [6, 11, 17–27]. The flowchart describing the process of this study selection was shown in Fig. 1. Studies were mainly concentrated in Asia, while only two articles in Europe were enrolled. In addition, three articles referred to different EBV genotypes between GRC and control. Based on different clinicopathologic characteristics, related data were chosen to analyze. Heterogeneity of most analysis in this study was small, so the fixed-model method was the main choice.

### EBV infection in GRC and CGC

**Fig. 2:**
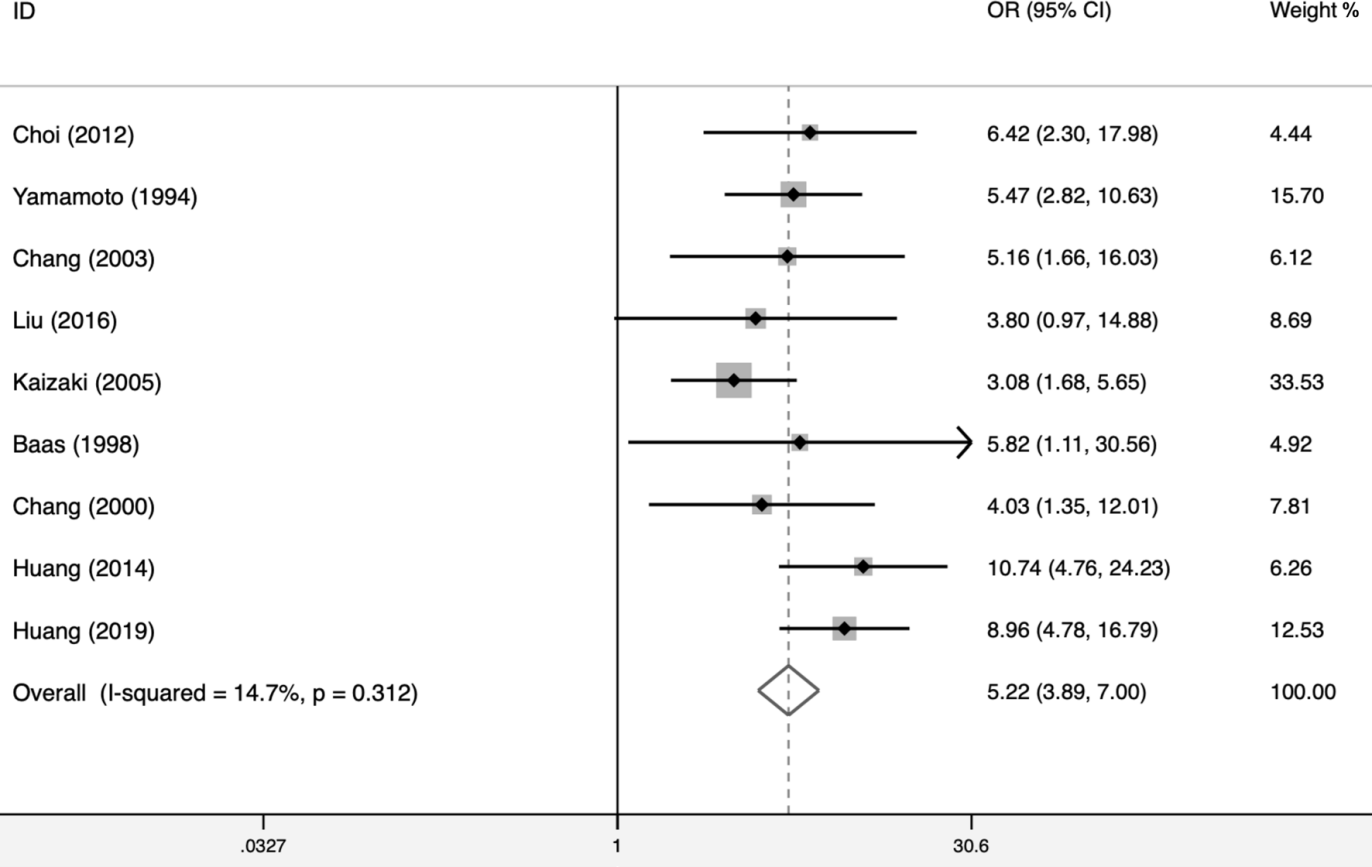
Compared to infection rate in CGC, the risk of EBV infection rate in the GRC was 5.22 times (OR = 5.22, 95% CI 3.89–7.00, I^2^ = 14.7%, P = 0.312)

Nine articles reported data about EBV infection in GRC and CGC. The main characteristics were listed in Table 1. The total EBV infection rate in GRC was 25.14% (91/362), while it was 2.25% (348/15433) in CGC. Compared to infection rate in CGC, the risk of EBV infection rate in the GRC was 5.22 times (OR = 5.22, 95% CI 3.89–7.00, I^2^ = 14.7%, P = 0.312) (Fig. 2). It could be seen that EBV plays a more important role in the pathogenesis of GRC than CGC.

**Table 1:** The main characteristics about EBV infection in GRC and CGC

Author	Country	Year	GRC, EBV+	GRC, EBV-	CGC, EBV+	CGC, EBV-	OR
Choi	Korea	2012	4	106	62	10,556	6.42
Yamamoto	Japan	1994	13	35	116	1709	5.47
Chang	Korea	2003	5	21	12	260	5.16
Liu	China	2016	10	36	3	41	3.80
Kaizaki	Japan	2005	18	60	46	472	3.08
Baas	Netherlands	1998	9	17	2	22	5.82
Chang	Korea	2000	5	21	17	288	4.03
Huang	Taiwan, China	2014	10	21	42	947	10.74
Huang	Taiwan, China	2019	17	45	48	1138	8.96

*EBV* Epstein–Barr virus, *GRC* gastric remnant carcinoma, *CGC* conventional gastric carcinoma, *OR* odds ratio

### Clinicopathologic characteristics between EBVaGRC and EBVnGRC

**Fig. 3:**
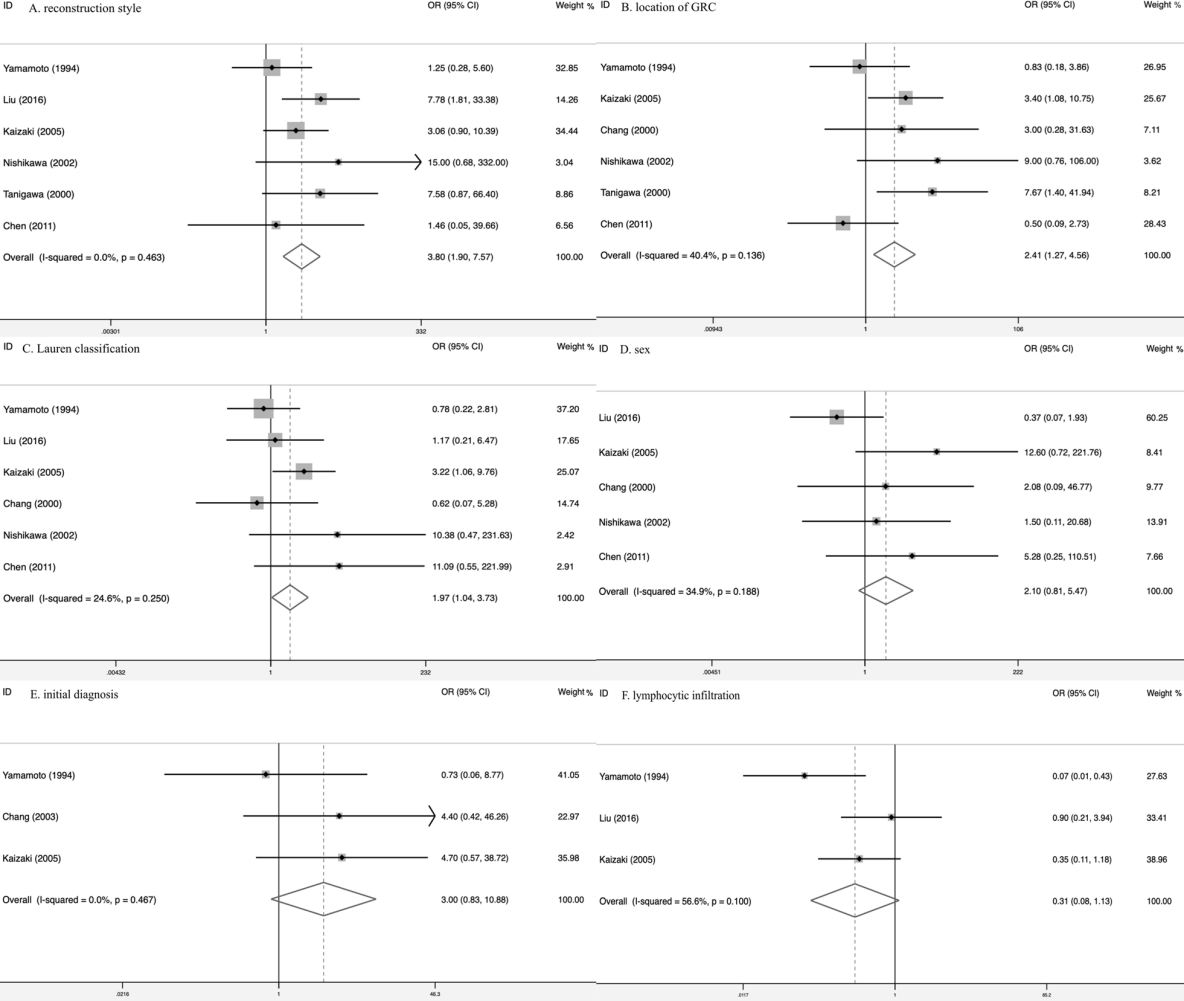
clinicopathologic characteristics between EBVaGRC and EBVnGRC (**a** reconstruction style; **b** location of GRC; **c** Lauren classification; **d** sex; **e** initial diagnosis; **f** lymphocytic infiltration)

According to the limited information provided in the articles, we analyzed clinicopathologic characteristics on reconstruction style after surgery (6 studies), initial diagnosis (3 studies), location of GRC (6 studies), Lauren classification (6 studies), sex of patients (5 studies), lymphocytic infiltration (3 studies). The main results were listed in Additional file 1: Table S1–Table S6. Reconstruction style, location of GRC and Lauren classification had statistic difference. The amount of Billroth-II was 3.8 times in EBVaGRC that of EBVnGRC (OR = 3.80, 95% CI 1.90–7.57, I^2^ = 0, P = 0.463) (Fig. 3a). Compared to EBVnGRC, EBVaGRC was 2.41 times more likely to become cancerous in the anastomotic site (OR = 2.41, 95% CI 1.27–4.56, I^2^ = 40.4%, P = 0.136) (Fig. 3b). In addition, for Lauren classification, diffuse type was more common in EBVaGRC, which was 1.97 times compared to EBVnGRC (OR = 1.97, 95% CI 1.04–3.73, I^2^ = 24.6%, P = 0.25) (Fig. 3c). However, initial diagnosis (OR = 3.0, 95% CI 0.83–10.88, I^2^ = 0, P = 0.467), sex (OR = 2.1, 95% CI 0.81–5.47, I^2^ = 34.9%, P = 0.188) and lymphocytic infiltration (OR = 0.31, 95% CI 0.08–1.13, I^2^ = 56.6%, P = 0.10) had no statistic difference between EBVnGRC and EBVaGRC (Fig. 3d–f).

### EBV genotypes between GRC and control

**Fig. 4:**
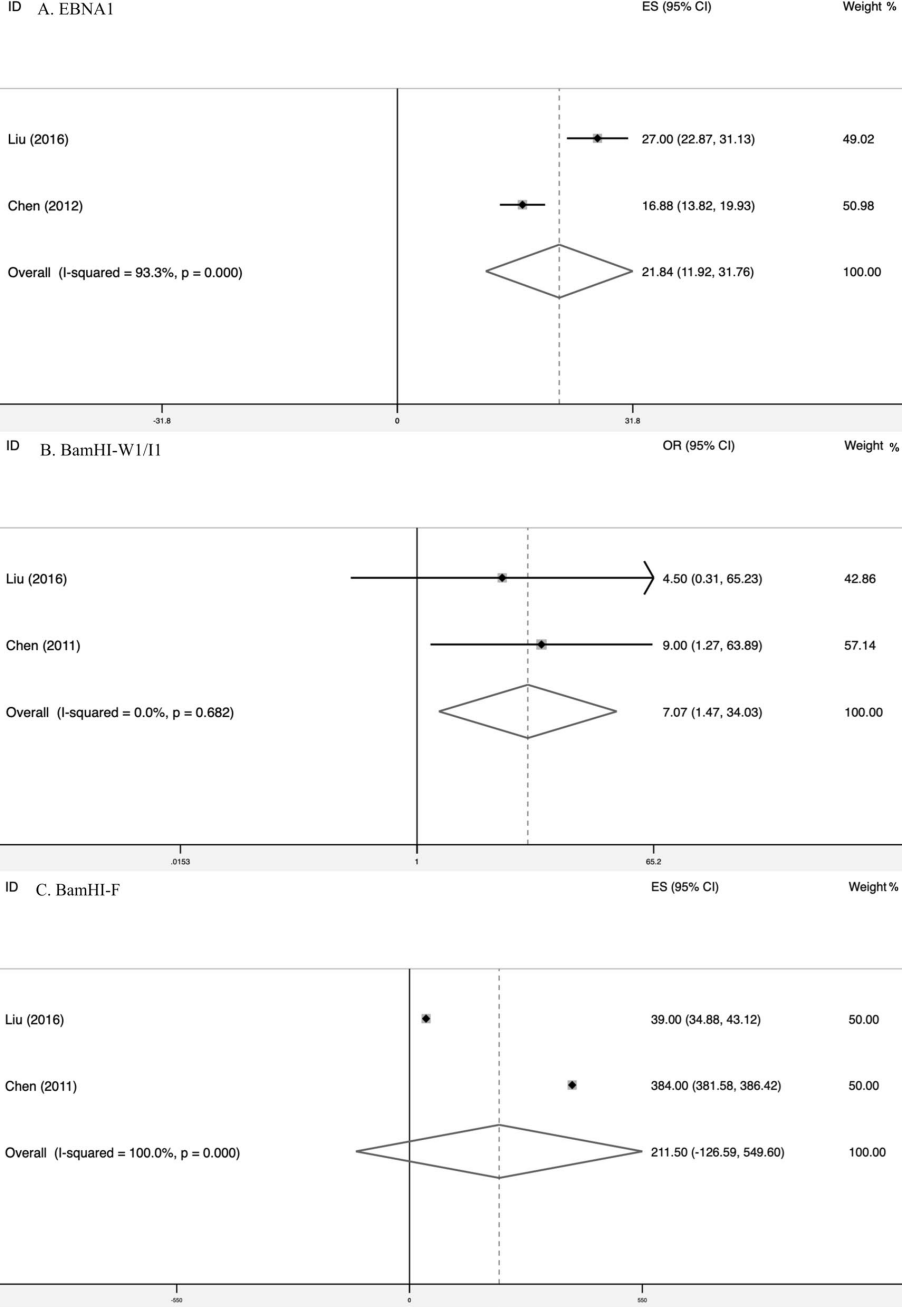
the difference of ENBA1, BamHI-W1/I1 and BamHI-F between EBVaGRC and control

Three articles referred EBV genotypes, which included EBNA1, EBNA2, EBNA3C, 30 bp deletion in LMP1, BamHI-F and BamHI-W1/I1. Of these, EBNA1, 30 bp deletion in LMP1, BamHI-F and BamHI-W1/I1 were reported by two articles. The main characteristics were listed in Table 2. EBNA2 and EBNA3C were no statistic difference between GRC and control reported by one article respectively (*P *= 0.624, *P* = 0.159, respectively). By meta-analysis, we found that “V-val” subtype of EBNA1 (OR = 21.84, 95% CI 11.92–31.76, I^2^ = 93.3%, P < 0.001) and “C” subtype of BamHI-W1/I1 (OR = 7.07, 95% CI 1.47–34.03, I^2^ = 0, P = 0.682) occupied larger proportion in GRC than control (Fig. 4). Nevertheless, subtype of BamHI-F had no difference between GRC and control (Fig. 4).

**Table 2:** The main characteristics of EBV genotypes between GRC and control

Genotype	Author	Year	Subtype			
EBNA1			Case “V-val” (B95.8, 109409)	Case “V-leu” (B95.8, 109408 ~ 109410)	Control “V-val” (B95.8, 109409)	Control “V-leu” (B95.8, 109408 ~ 109410)
	Liu	2016	9	0	3	0
	Chen	2012	0	4	15	9
EBNA2			Case “1” (497 bp)	Case “2” (-)	Control “1” (497 bp)	Control “2” (-)
	Liu	2016	13	0	3	0
EBNA3C			Case “A” (153 bp)	Case “B” (246 bp)	Control “A” (153 bp)	Control “B” (246 bp)
	Chen	2011	8	0	12	4
BamHI-F			Case “F” (198 bp)	Case “f” (127 + 71 bp)	Control “F” (198 bp)	Control “f” (127 + 71 bp)
	Liu	2016	13	0	3	0
	Chen	2011	8	1	16	3
BamHI-W1/I1			Case “C” (205 bp)	Case “D” (130 + 75 bp)	Control “C” (205 bp)	Control “D” (130 + 75 bp)
	Liu	2016	9	4	1	2
	Chen	2011	6	2	4	12
30 bp deletion in LMP1			Case “del” (189 bp)	Case “wt” (219 bp)	Control “del” (189 bp)	Control “wt” (219 bp)
	Liu	2016	12	1	3	0
	Chen	2011	8	0	–	–

*GRC* gastric remnant carcinoma

### Publication bias and sensitivity analysis

Begg’s test showed that there was no publication bias in this meta-analysis, including EBV infection in GRC and CGC (P = 0.917), reconstruction style (P = 0.851), initial diagnosis (P = 0.296), location of GRC (P = 0.707), Lauren classification (P = 0.851), sex of patients (P = 0.462) and lymphocytic infiltration (P = 0.602) (Additional file 2). In addition, sensitivity analysis results showed that some results of dichotomous variable including EBV infection comparisons and clinicopathologic characteristics comparisons were fluctuant, but they were still stable and reliable (Additional file 3).

## Discussion

In this study, we firstly reported that EBV infection rate in the GRC was higher than CGC. EBVaGRC had higher rate of Billroth-II, carcinoma in anastomotic site and diffuse type (Lauren classification) compared to EBVnGRC, but it is not associated with sex, initial diagnosis and lymphocytic infiltration. In addition, EBV genome polymorphisms analysis showed that expression of “V-val” subtype of EBNA1 and “C” subtype of BamHI-W1/I1 were higher in EBVaGRC than control. We comprehensively analyzed EBV infection and genome polymorphisms on GRC through systematic review and meta-analysis for the first time.

Up until now, the role of EBV in carcinogenesis of the gastric remnant or stomach is not completely understood. It has been suggested that EBVaGC have distinct molecular characteristics, as well as clinicopathological characteristics [5]. The distinguish in EBVaGRC was also found in this study. The loss of *p*16 protein [28], or over-expression of bcl-2 [29] was detected in EBV-infected tumors or EBVaGC, which may be related to the pathogenesis of GRC. In addition, Imai et al. revealed that EBV is uniformly present in all carcinoma cells of GRC, but nearly negative in normal epithelium or stroma [3]. The findings indicated that EBV infection may influence carcinogenesis, perhaps triggering a clonal expansion of infected cells [3].

In the study, three clinicopathological factors were observed related to EBVaGRC. EBVaGRC was more likely to occur in anastomotic sites and after Billroth-II reconstruction. The changes of anatomical circumstances may be the main factor. The reflux of bile and pancreatic juice is more common in Billroth-II reconstruction, which can directly change the physiological environment and pH-value of the gastric remnant. These could act as a cofactor mediating EBV infection of the epithelial cells or facilitate EBV entering the mucosa epithelia, for instance by inducing fusion of EBV carrying B cells and epithelial cells. In addition, more diffuse type of GRC can be found with EBV infection. This type of cancer usually presents shorter duration and worse prognosis compared with the intestinal type [30]. Therefore, EBV infection may aggravate the severity of the cancer. Although some articles have found a correlation between sex, initial diagnosis, lymphocytic infiltration and EBVaGRC, the results of this study revealed no correlation by data Integration.

In addition, we paid attention to survival rate of GRC, but no articles reported it. Liu et al. reported that EBV had a favorable impact on GC patient’s survival, especially in an Asian population [31]. In GRC, whether EBV can influence the survival rate, we need more studies to support evidence.

We also found “V-val” subtype of EBNA1 and “C” subtype of BamHI-W1/I1 were higher expressed in EBVaGRC. These two subtypes may be closely related to oncogenesis and be as potential disease predictor. It is known that EBNA1 can induce genomic instability and reactive oxygen species (ROS)-mediated DNA damage response [32], which may act as a potential oncogene [33]. EBNA1 contains two prototypes (P-ala and P-thr) and three variants (V-pro, V-leu and V-val) [34]. It was reported that in EBV-associated neoplasms, “V-val” was predominant in individuals from Asian regions [35], while NPC, EBVaGC and nasal NK/T cell lymphoma are more prevalent in this area [36]. This phenomenon may suggest that “V-val” subtype might be more aggressive than other subtypes. The possible reason was that most of amino acid alterations of “V-val” subtype (codons 439, 487, 499, 502, 524, 528 and 533) were located in the function domains of EBNA1, which contained DNA binding domain (aa 459,487), dimerization domain (aa 501,532) and transactivation domain (aa 450,641) [26]. Thus “V-val” subtype was more likely to cause changes of EBNA1’s function. In addition, previous studies revealed that “C” subtype prevails in NPC patients from Southern China where incidence of NPC is high. It was suggested that the presence of “C” subtype is also associated with oncogenesis. Therefore, “V-val” subtype and “C” subtype can be tested to predict high risk of EBVaGRC.

Although we have achieved some encouraging results, this article still had some defects. First, included articles had limitations due to article source and type. Articles were mainly from the Asian region and had no global representation. In addition, we can only clarify the relationship between EBV and GRC, but there was not enough evidence of causality. Enough randomized controlled trials (RCTs) were needed to clarify the causality. Second, the credibility of results was weakened because of insufficient data, especially analysis of EBV genome polymorphisms. Third, since the ORs of the included studies were crude ORs which were calculated by authors, there was of some heterogeneity. This part of heterogeneity cannot be ruled out, because we cannot obtain data directly from authors. And next, in this study, it was not certain that GRC originated from a recurrence of the previous GC or a “de novo” EBV-related GC. Homology of cancer is also crucial for patient treatment and follow-up. Finally, we didn’t know whether EBV eradication can change the process of GRC. If EBV eradication weakened oncogenesis of gastric remnant, it will provide strong evidence that EBV is an important causative agent to GRC. Such articles were really needed.

## Conclusion

In conclusion, Compared to CGC, EBV infection rate in the GRC was higher. Further analysis showed that the ratio of Billroth-II, carcinoma in anastomotic site and diffuse type (Lauren classification) were higher in EBVaGRC, which were clinicopathologic characteristics of EBVaGRC. Through analysis of EBV genome polymorphisms, “V-val” subtype of EBNA1 and “C” subtype of BamHI-W1/I1 may become predictor of EBVaGRC, and genetic test of these two can guide us to conduct early GRC intervention.

## Supplementary information

**Additional file 1.** The main results of clinicopathologic characteristics on reconstruction style after surgery, initial diagnosis, location of GRC, Lauren classification , sex of patients, and lymphocytic infiltration.

**Additional file 2.** Begg’s test of EBV infection and clinicopathologic characteristics.

**Additional file 3.** Sensitivity analysis results of EBV infection comparisons and clinicopathologic characteristics comparisons.

## Data Availability

The datasets used and/or analysed during the current study are available from the corresponding author on reasonable request.

## Abbreviations

EBV: Epstein–Barr virus
GRC: Gastric remnant carcinoma
CGC: Conventional gastric carcinoma
EBVaGC: EBV-associated-gastric carcinoma
EBVaGRC: EBV-associated-gastric remnant carcinoma
EBVnGRC: EBV-negative-gastric remnant carcinoma
