# Supplementary material for: Epstein–Barr virus infection and genome polymorphisms on gastric remnant carcinoma: a meta-analysis
Source: Cancer Cell Int. 2020 Aug 18;20:401. doi: 10.1186/s12935-020-01498-z (PMC7437139; doi:10.1186/s12935-020-01498-z)
Supplement: Supplementary file 1 — Additional file 1. The main results of clinicopathologic characteristics on reconstruction style after surgery, initial diagnosis, location of GRC, Lauren classification , sex of patients, and lymphocytic infiltration. [file 12935_2020_1498_MOESM1_ESM.pdf]

Table S1: The main characteristics about EBV infection in Billroth-I and Billroth-II

Author	Year	Country	Billroth-I EBV+	Billroth-I EBV-	Billroth-II EBV+	Billroth-II EBV-	OR
Yamamoto	1994	Japan	3	10	9	24	0.80
Liu	2016	China	3	28	10	12	0.13
Kaizaki	2005	Japan	4	28	14	32	0.33
Nishikawa	2002	Sweden	0	5	7	5	-
Tanigawa	2000	Japan	1	16	9	19	0.13
Chen	2011	China	0	1	8	17	-

EBV: Epstein-Barr virus

OR: odds ratio

Table S2: The main characteristics about EBV infection in benign and malignant tumor

Author	Year	Country	benign EBV+	benign EBV-	malignant EBV+	malignant EBV-	OR
Yamamoto	1994	Japan	12	33	1	2	0.73
Chang	2003	Korea	4	10	1	11	4.40
Kaizaki	2005	Japan	17	47	1	13	4.70

EBV: Epstein-Barr virus

OR: odds ratio

Table S3: The main characteristics about EBV infection in anastomotic stoma or not

Author	Year	Country	Anastomotic stoma, EBV+	Anastomotic stoma, EBV-	Other, EBV+	Other, EBV-	OR
Yamamoto	1994	Japan	10	28	3	7	0.83
Kaizaki	2005	Japan	13	26	5	34	3.40
Chang	2000	Korea	4	12	1	9	3.00
Nishikawa	2002	Sweden	6	4	1	6	9.00
Tanigawa	2000	Japan	8	12	2	23	7.67
Chen	2011	China	4	12	4	6	0.50

EBV: Epstein-Barr virus

OR: odds ratio

Table S4: The main characteristics about EBV infection under different Lauren classification

Author	Year	Country	Intestinal, EBV+	Intestinal, EBV-	Diffuse, EBV+	Diffuse, EBV-	OR
Yamamoto	1994	Japan	6	14	7	21	1.29
Liu	2016	China	2	7	11	33	0.86
Kaizaki	2005	Japan	6	37	12	23	0.31
Chang	2000	Korea	2	8	2	13	1.63
Nishikawa	2002	Sweden	0	4	7	6	-
Chen	2011	China	0	7	8	11	-

EBV: Epstein-Barr virus

OR: odds ratio

Table S5: The main characteristics about EBV infection in male and female

Author	Year	Country	Male, EBV+	Male, EBV-	Female, EBV+	Female, EBV-	OR
Liu	2016	China	10	36	3	4	0.37
Kaizaki	2005	Japan	18	45	0	15	-
Chang	2000	Korea	5	18	0	3	-
Nishikawa	2002	Sweden	6	8	1	2	1.50
Chen	2011	China	8	14	0	4	-

EBV: Epstein-Barr virus

OR: odds ratio

Table S6: The main characteristics about EBV infection under different lymphocytic infiltration

Author	Year	Country	Mild, EBV+	Mild, EBV-	Severe, EBV+	Severe, EBV-	OR
Yamamoto	1994	Japan	7	33	6	2	0.07
Liu	2016	China	3	10	10	30	0.90
Kaizaki	2005	Japan	12	51	6	9	0.35

EBV: Epstein-Barr virus

OR: odds ratio
