# Supplementary material for: Epstein–Barr virus infection and genome polymorphisms on gastric remnant carcinoma: a meta-analysis
Source: Cancer Cell Int. 2020 Aug 18;20:401. doi: 10.1186/s12935-020-01498-z (PMC7437139; doi:10.1186/s12935-020-01498-z)
Supplement: Supplementary file 2 — Additional file 2. Begg’s test of EBV infection and clinicopathologic characteristics. [file 12935_2020_1498_MOESM2_ESM.pdf]

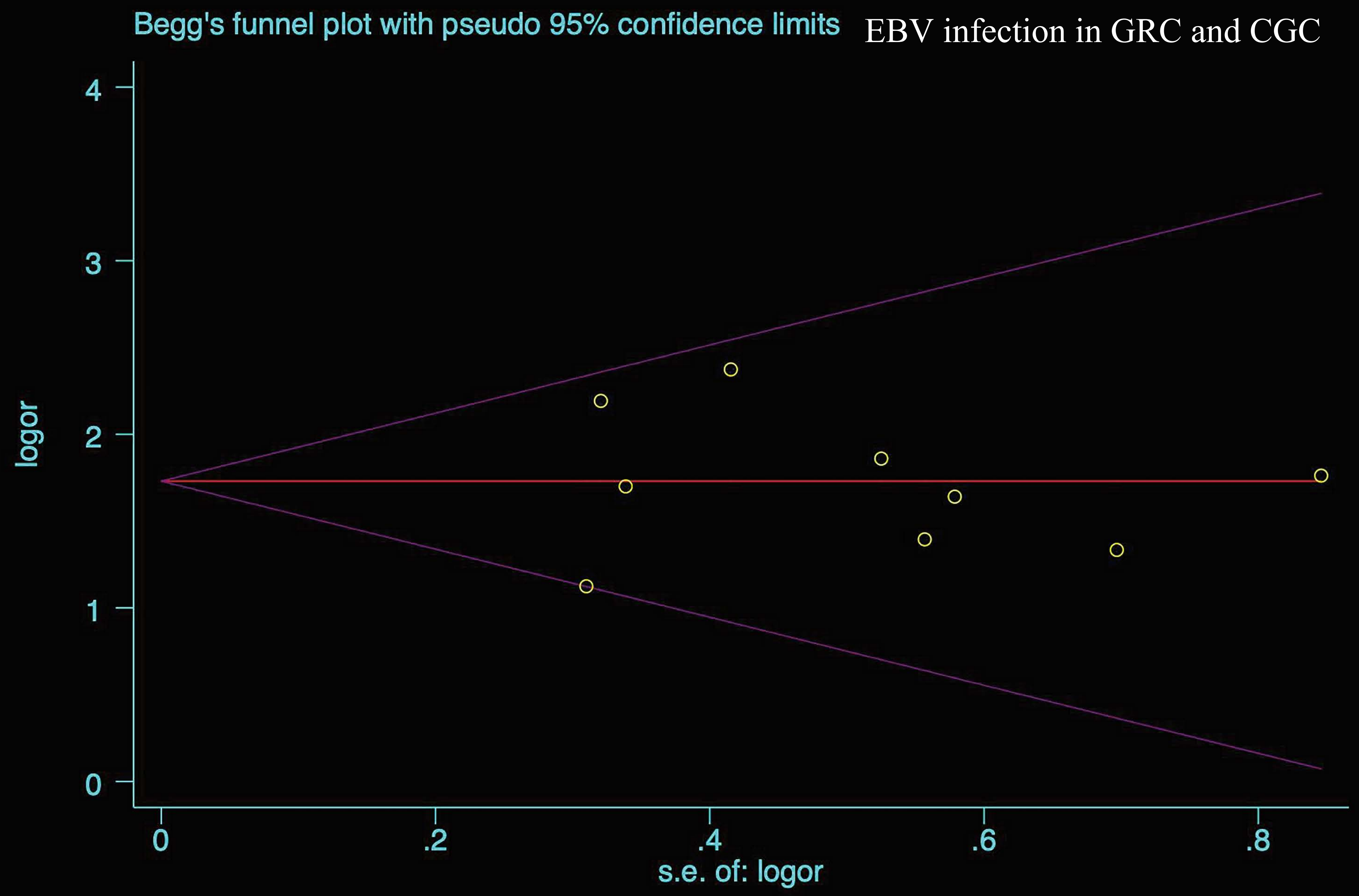

Begg's funnel plot with pseudo 95% confidence limits
EBV infection in GRC and CGC
logor
4
3
2
1
0
0
.2
.4
.6
.8
s.e. of: logor

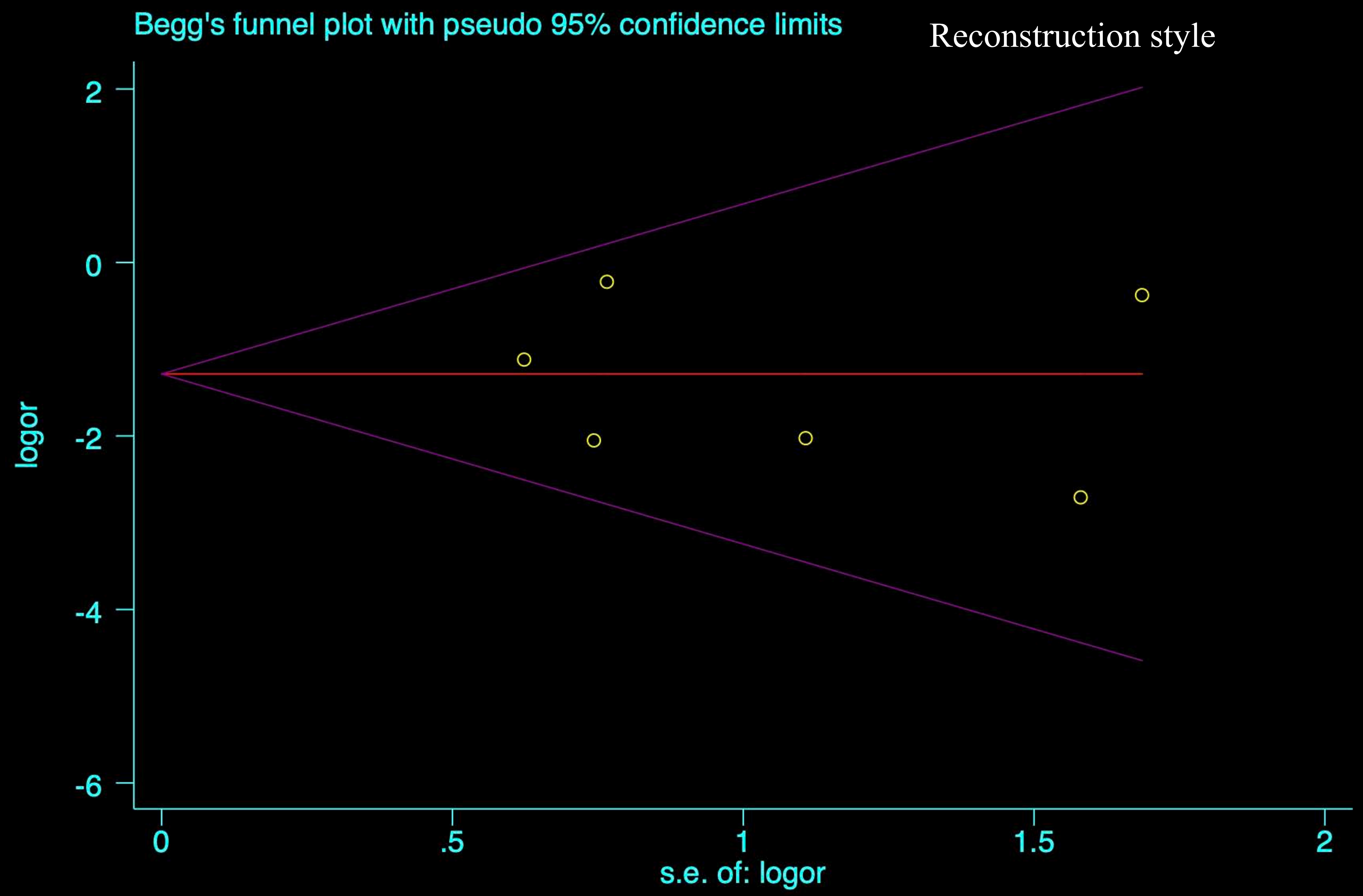

Begg's funnel plot with pseudo 95% confidence limits
Reconstruction style
2
0
-2
-4
-6
logor
0
.5
1
1.5
2
s.e. of: logor

Location of GRC

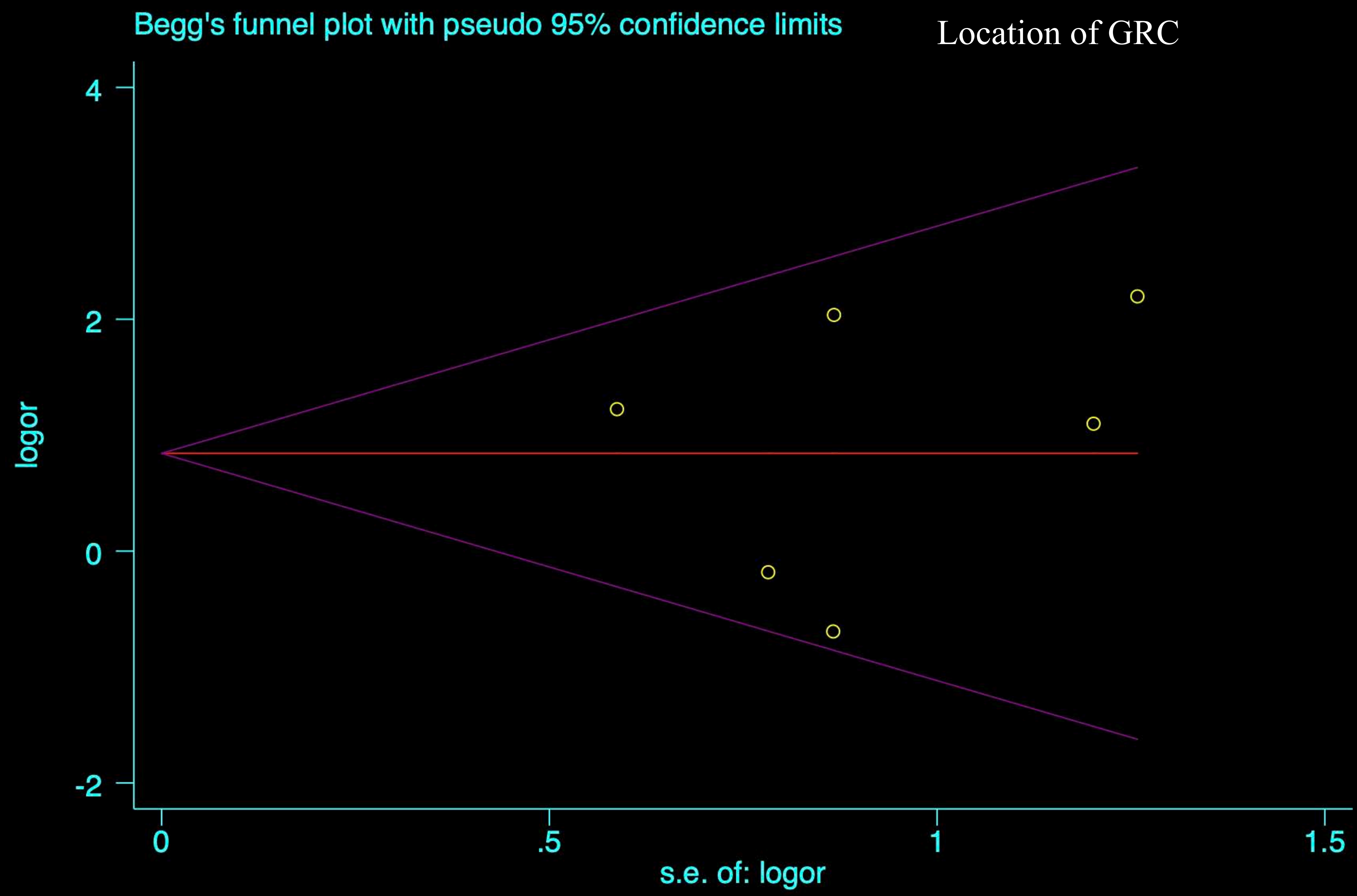

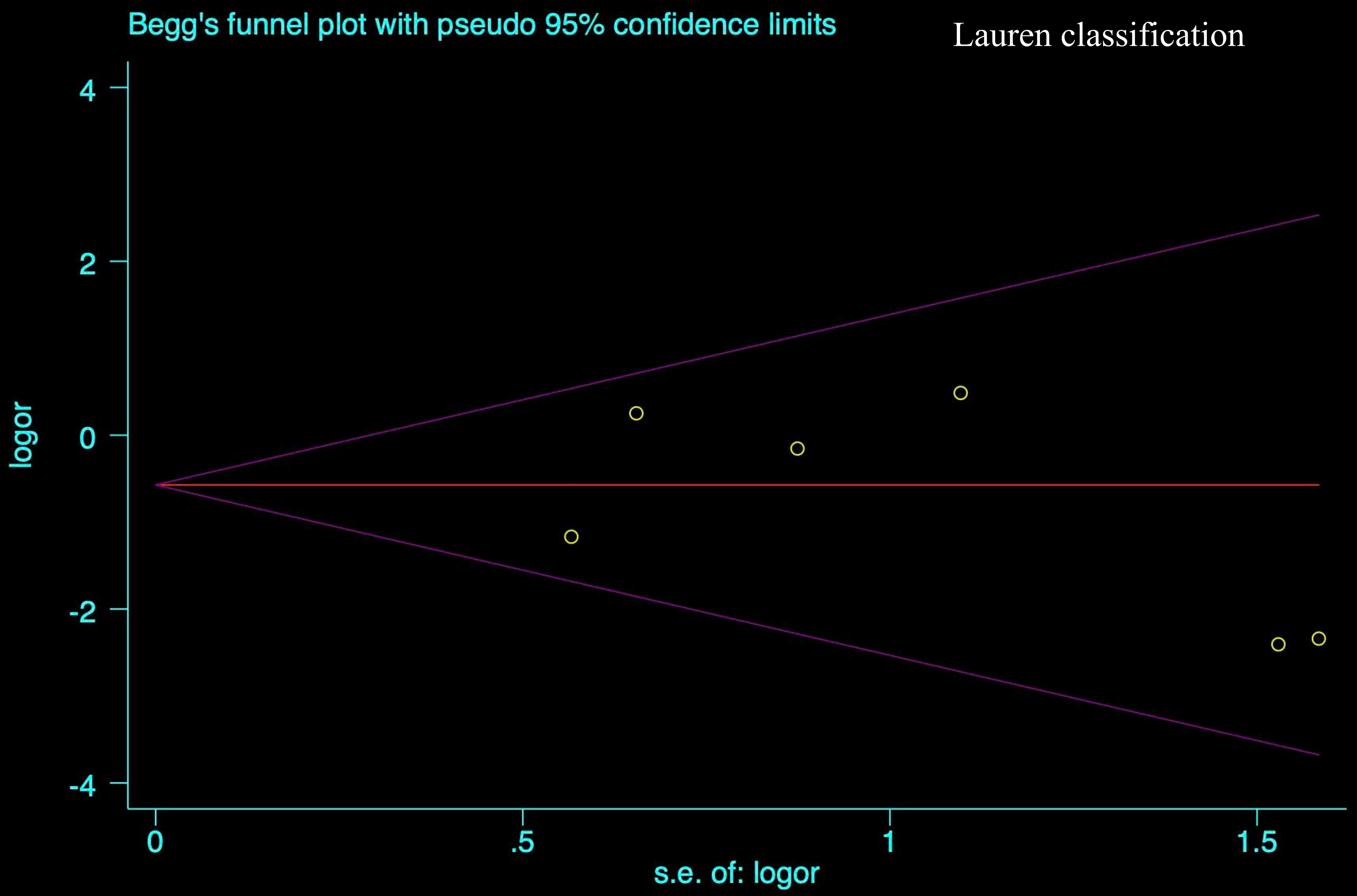

Begg's funnel plot with pseudo 95% confidence limits
Lauren classification
logor
4
2
0
-2
-4
0
.5
1
1.5
s.e. of: logor

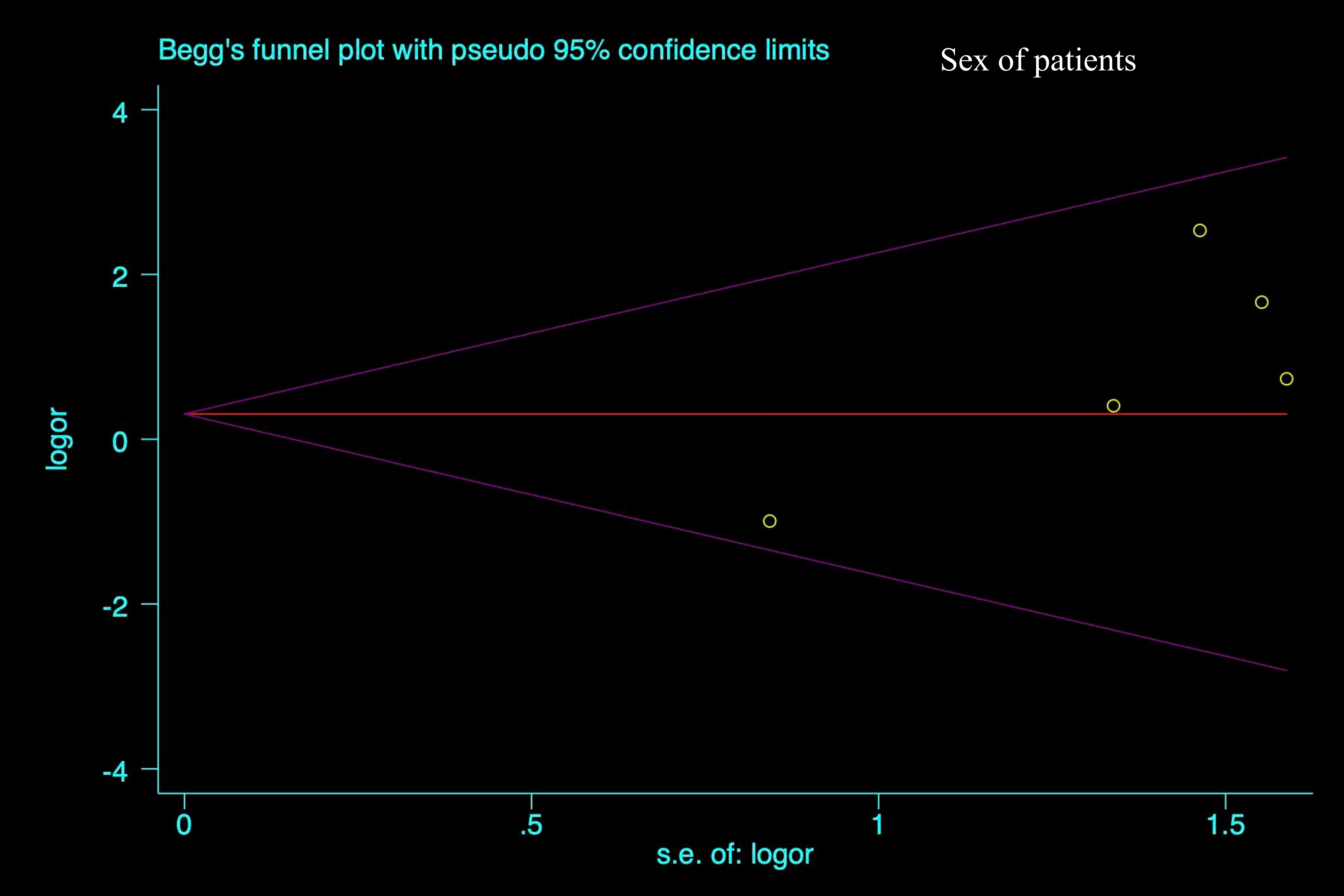
Begg's funnel plot with pseudo 95% confidence limits
Sex of patients
4
2
0
-2
-4
logor
0
.5
1
1.5
s.e. of: logor

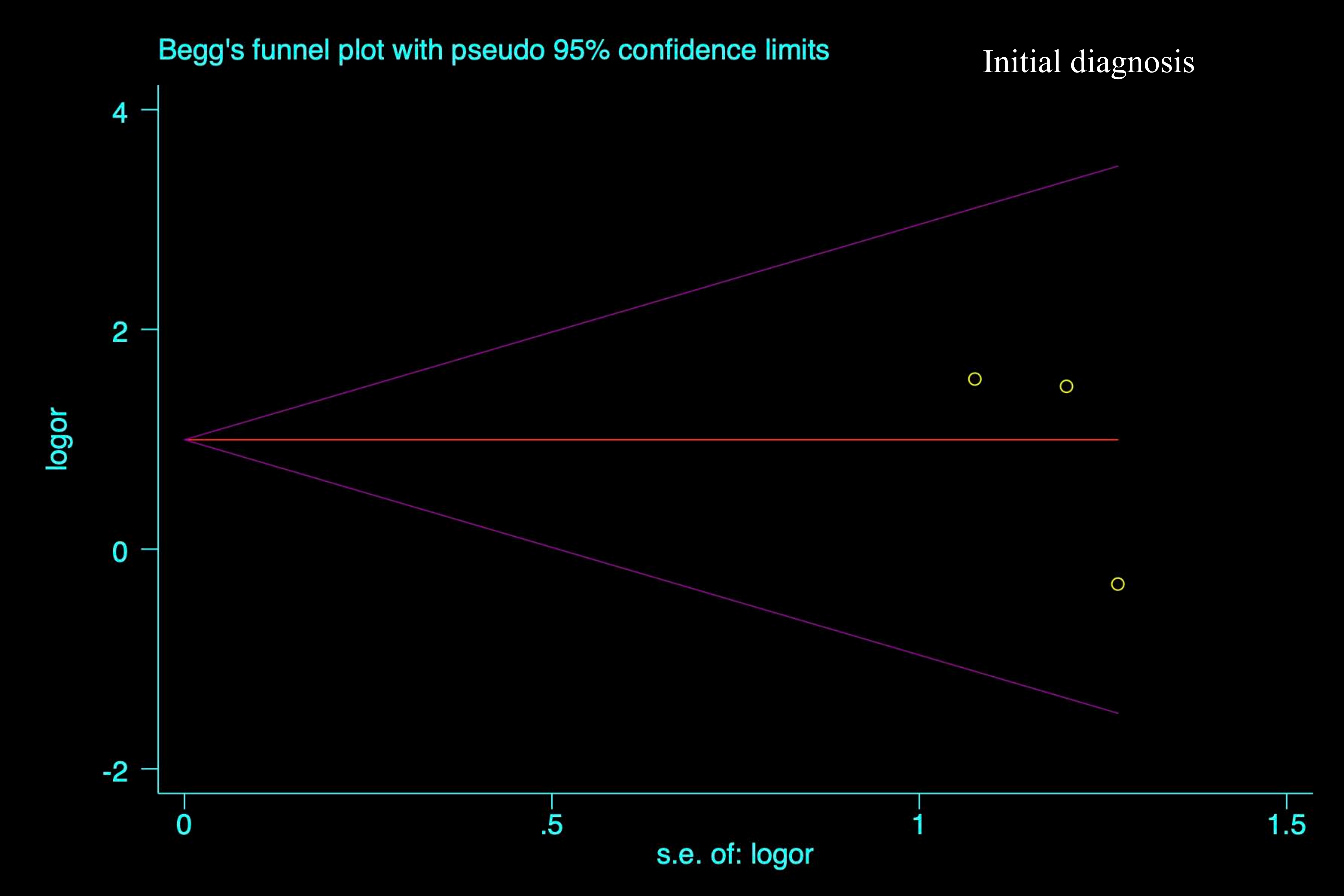
Begg's funnel plot with pseudo 95% confidence limits
Initial diagnosis
4
2
0
-2
logor
0
.5
1
1.5
s.e. of: logor

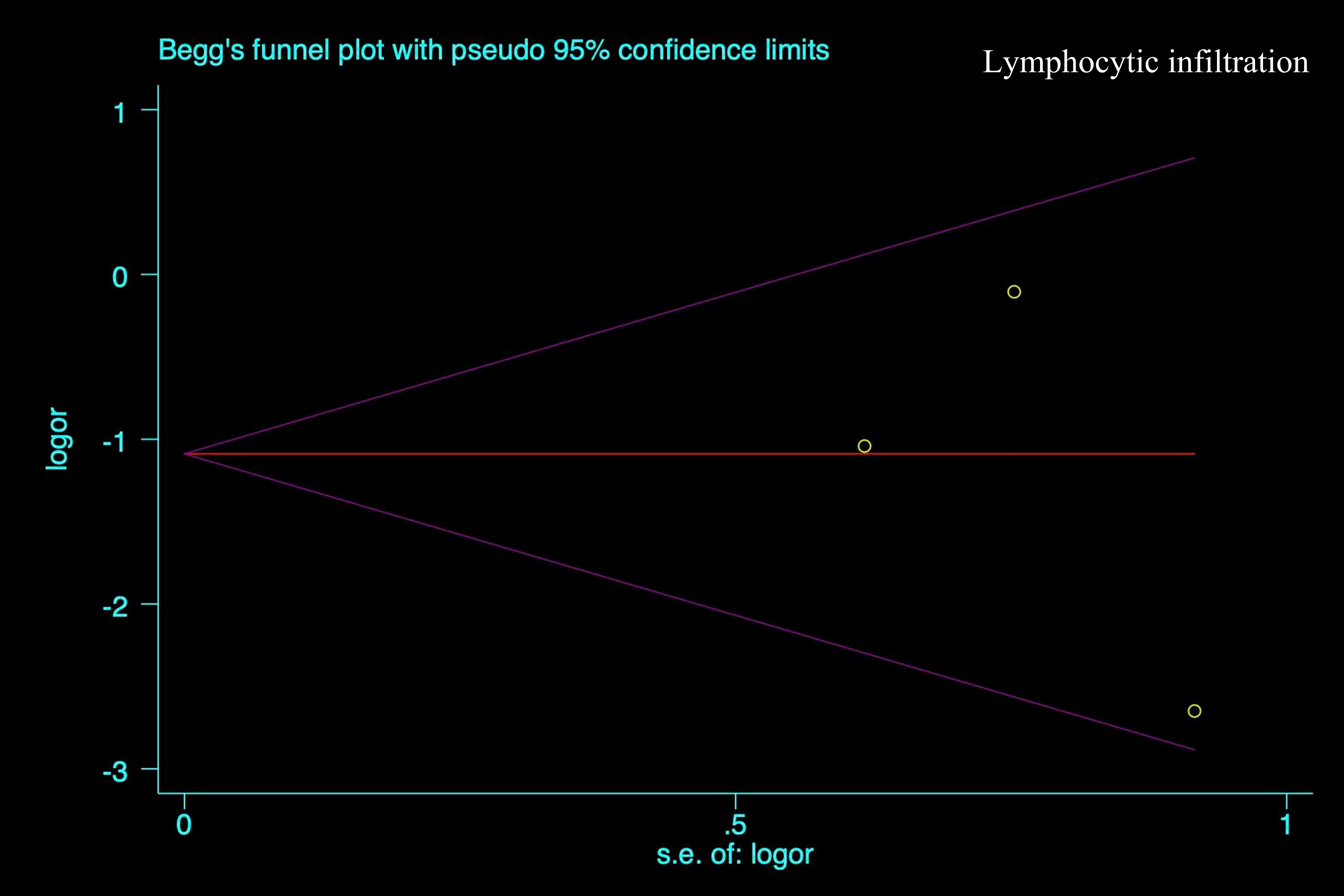
Begg's funnel plot with pseudo 95% confidence limits
Lymphocytic infiltration
1
0
-1
-2
-3
logor
0
.5
1
s.e. of: logor
