# Supplementary material for: Epstein–Barr virus infection and genome polymorphisms on gastric remnant carcinoma: a meta-analysis
Source: Cancer Cell Int. 2020 Aug 18;20:401. doi: 10.1186/s12935-020-01498-z (PMC7437139; doi:10.1186/s12935-020-01498-z)
Supplement: Supplementary file 3 — Additional file 3. Sensitivity analysis results of EBV infection comparisons and clinicopathologic characteristics comparisons. [file 12935_2020_1498_MOESM3_ESM.pdf]

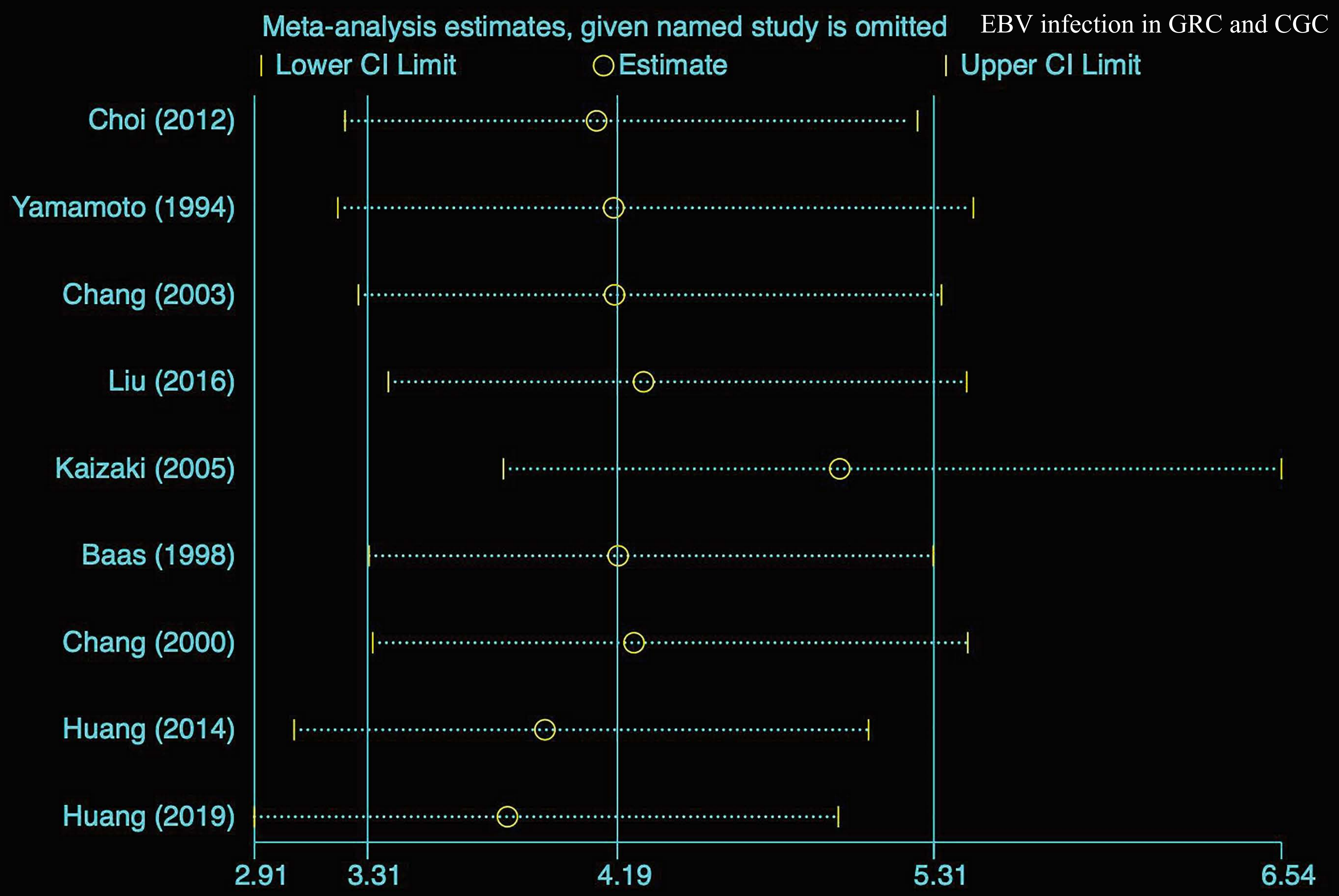

EBV infection in GRC and CGC
Meta-analysis estimates, given named study is omitted
Lower CI Limit
Estimate
Upper CI Limit
Choi (2012)
Yamamoto (1994)
Chang (2003)
Liu (2016)
Kaizaki (2005)
Baas (1998)
Chang (2000)
Huang (2014)
Huang (2019)
2.91
3.31
4.19
5.31
6.54

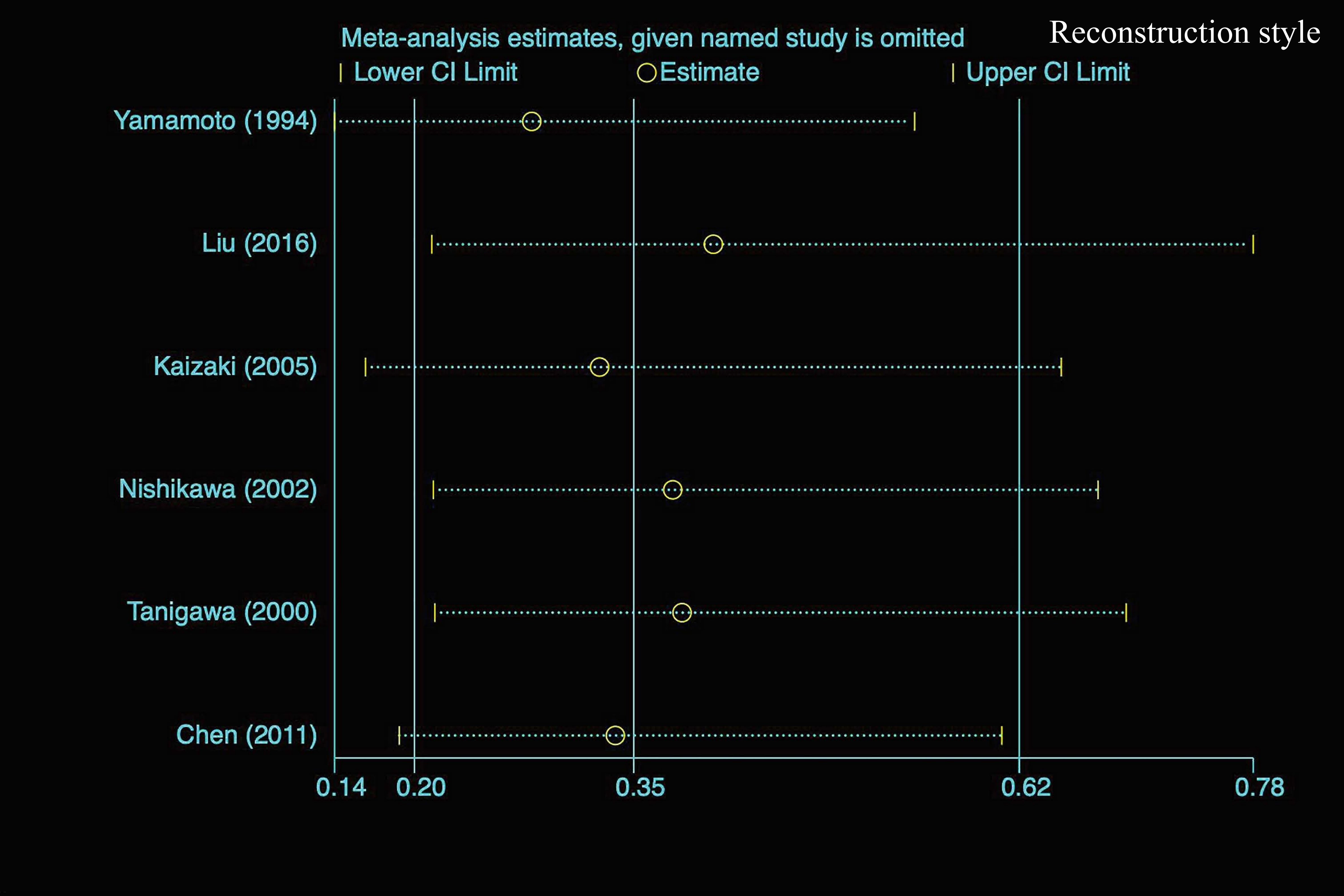

Reconstruction style
Meta-analysis estimates, given named study is omitted
Lower CI Limit
Estimate
Upper CI Limit
Yamamoto (1994)
Liu (2016)
Kaizaki (2005)
Nishikawa (2002)
Tanigawa (2000)
Chen (2011)
0.14
0.20
0.35
0.62
0.78

Location of GRC

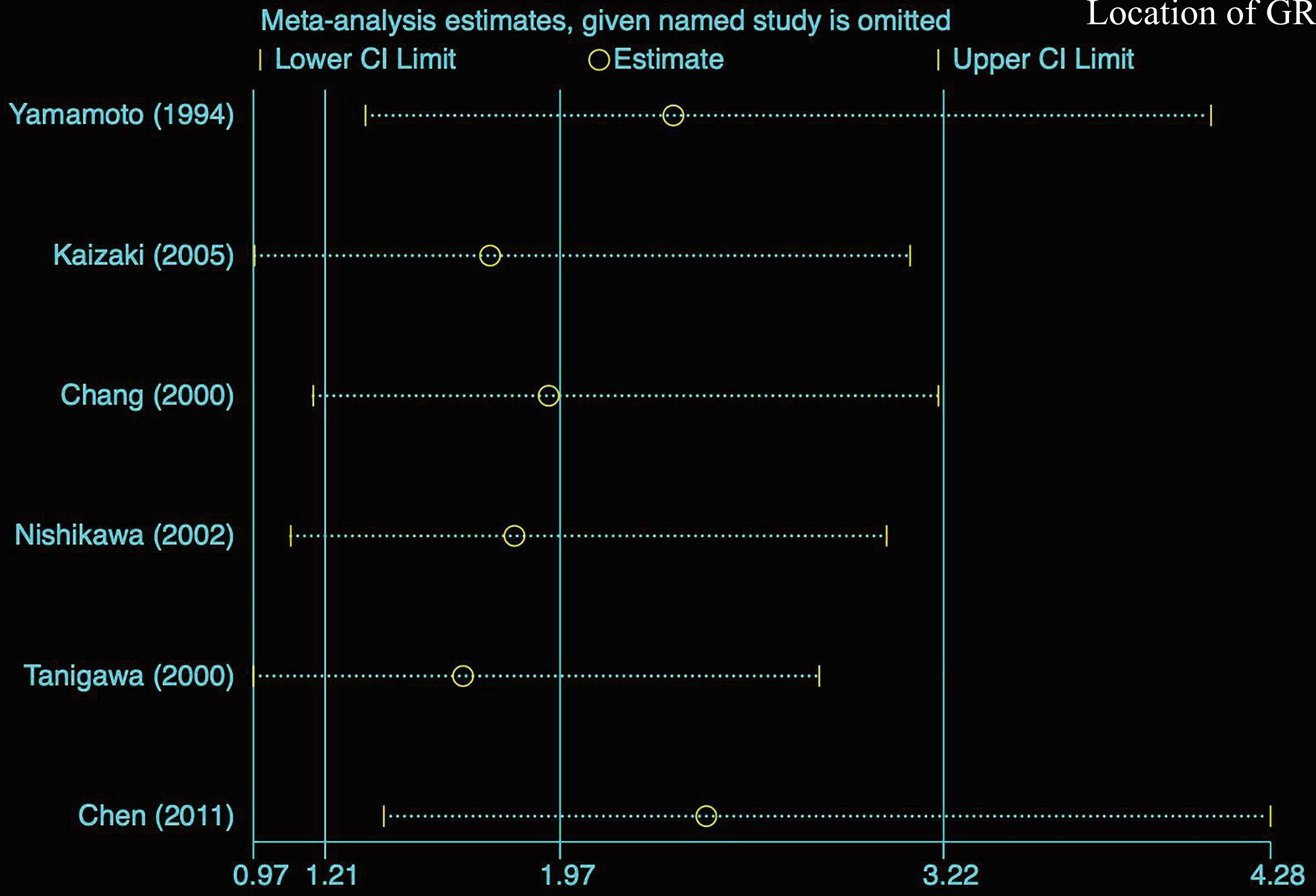

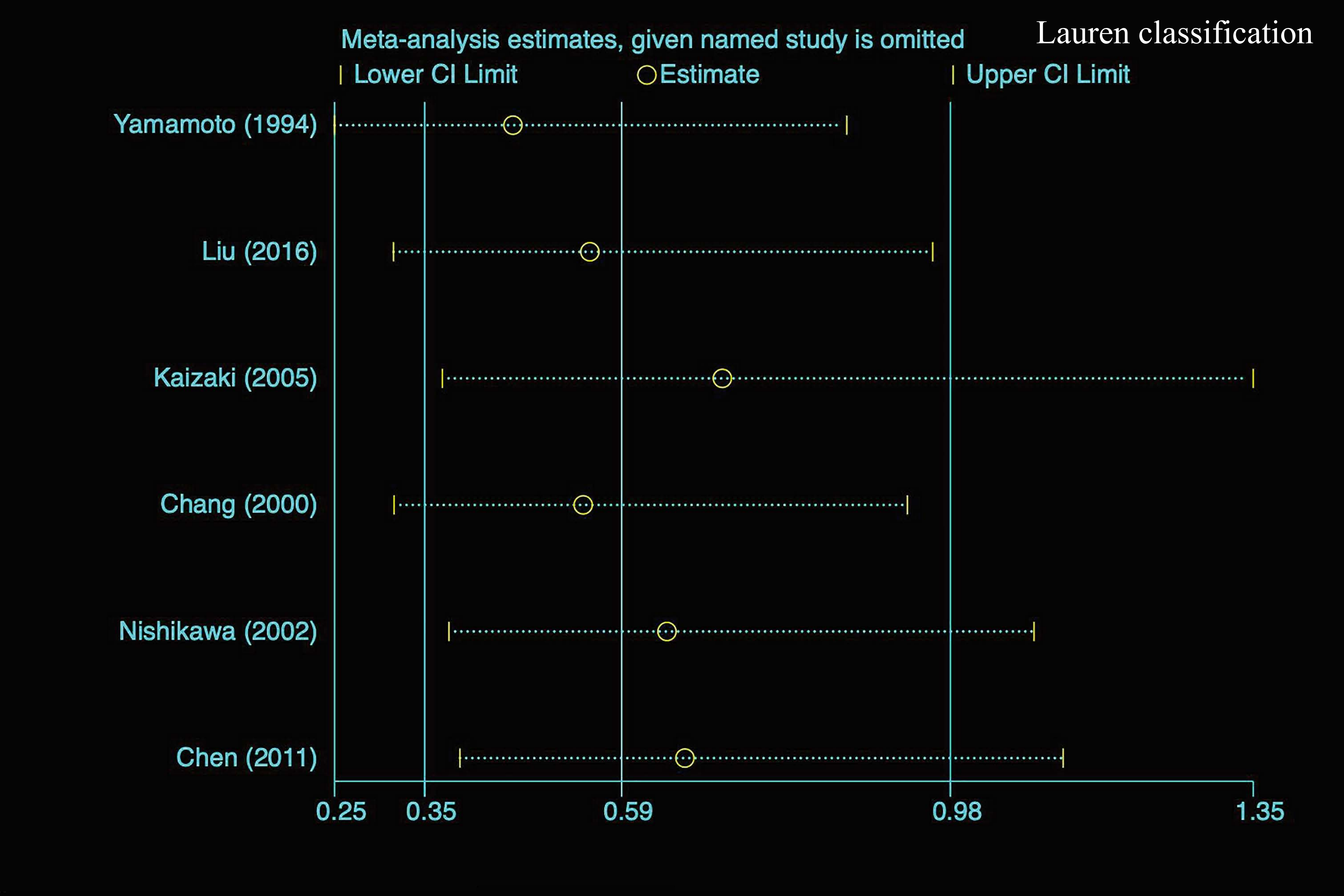

Lauren classification
Meta-analysis estimates, given named study is omitted
Lower CI Limit
Estimate
Upper CI Limit
Yamamoto (1994)
Liu (2016)
Kaizaki (2005)
Chang (2000)
Nishikawa (2002)
Chen (2011)
0.25
0.35
0.59
0.98
1.35

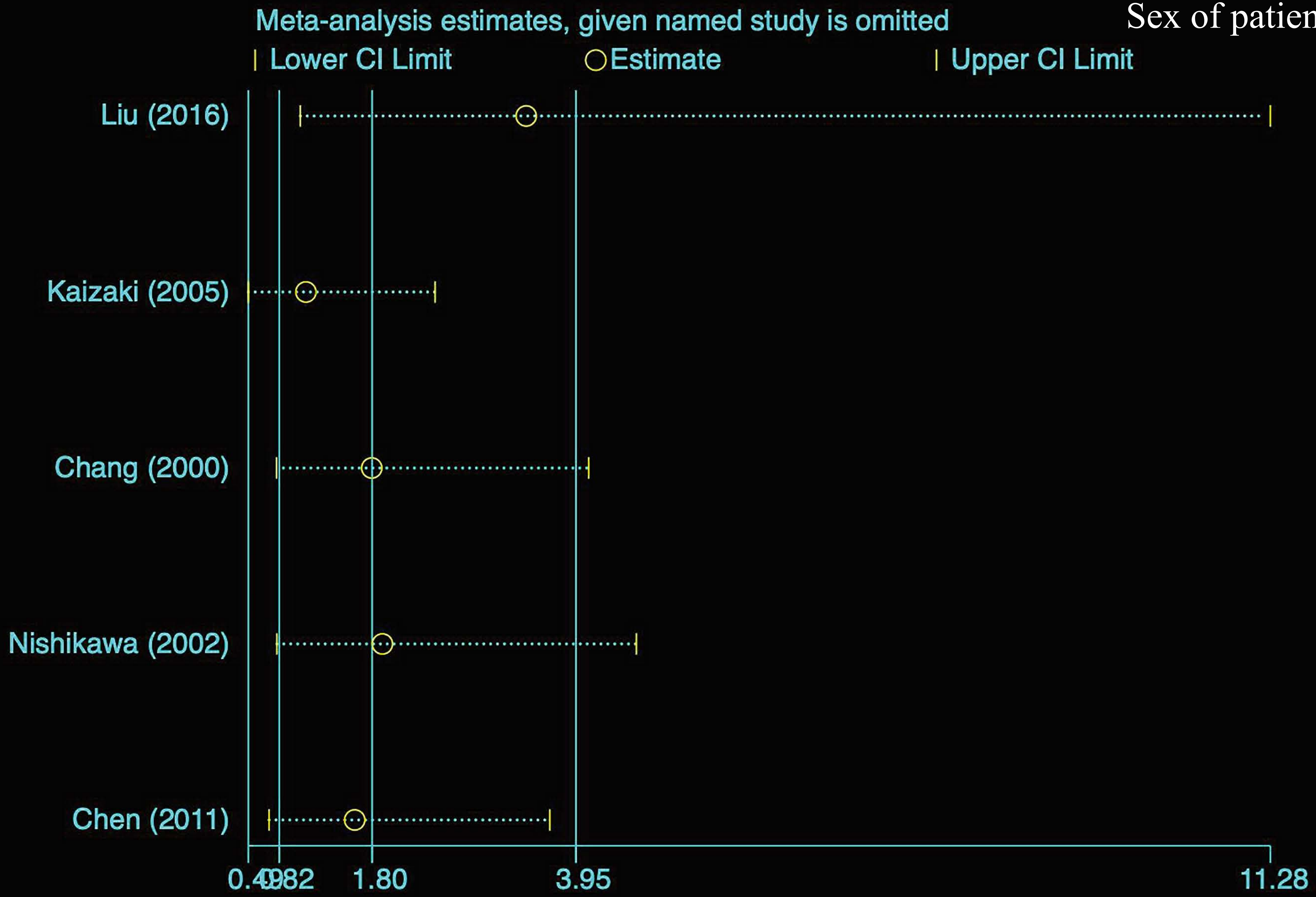

Sex of patients

Initial diagnosis

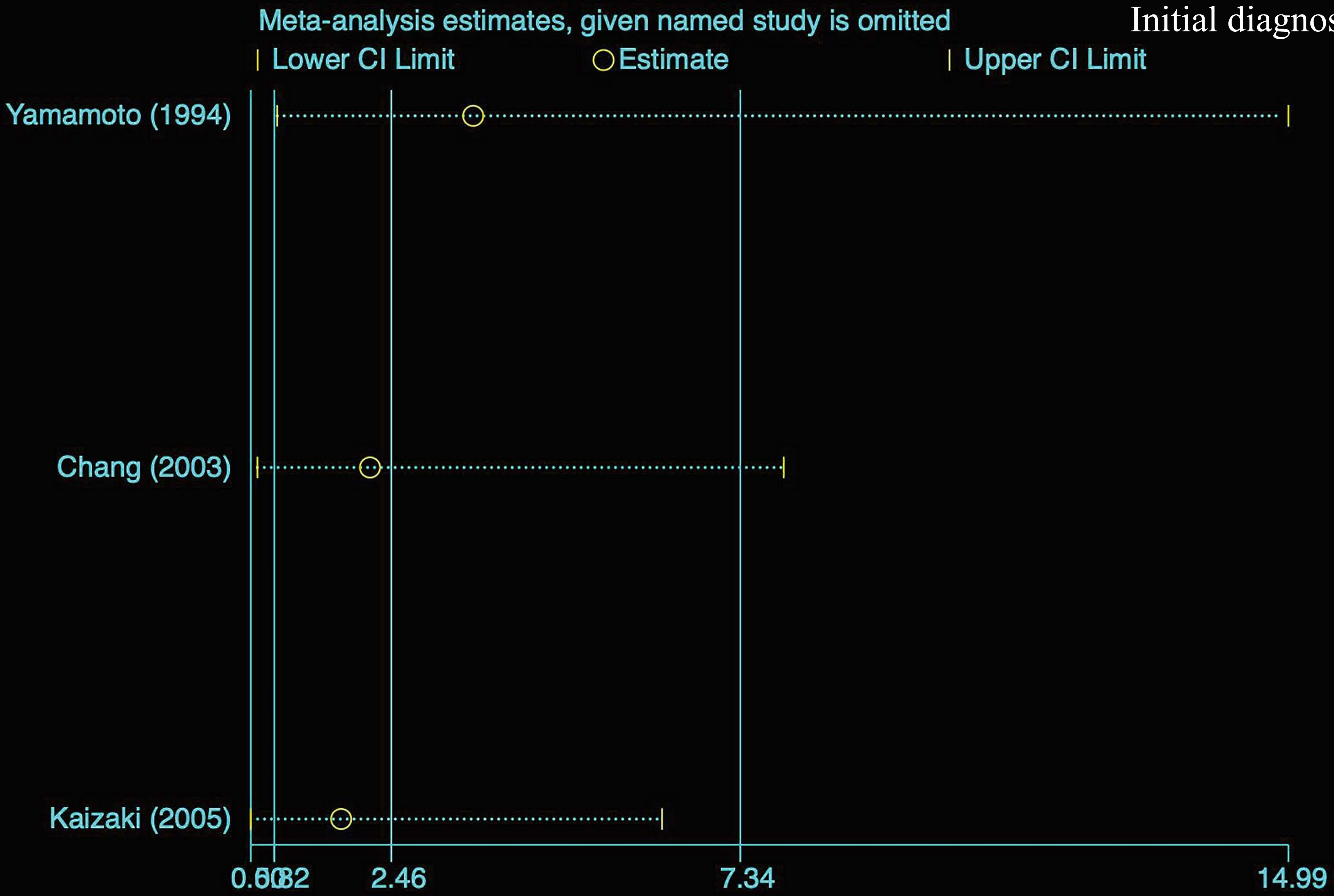

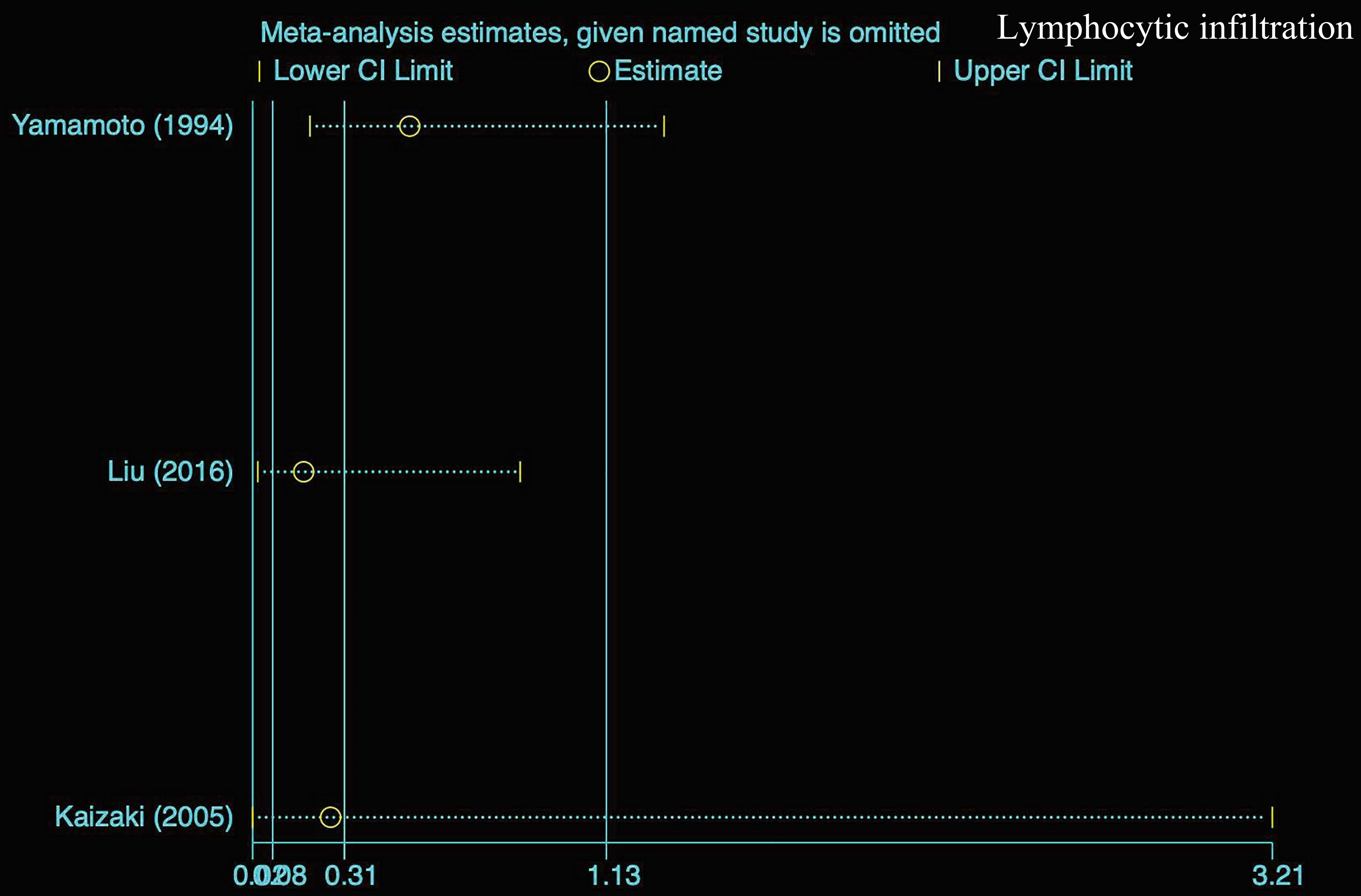

Lymphocytic infiltration
Meta-analysis estimates, given named study is omitted
Lower CI Limit
Estimate
Upper CI Limit
Yamamoto (1994)
Liu (2016)
Kaizaki (2005)
0.31
1.13
3.21
